# Machine learning nomograms for gastric cancer: addressing data limitations in SEER-based models

**DOI:** 10.1007/s00423-026-04014-5

**Published:** 2026-04-08

**Authors:** Amirhosein Naseri, Mohammad Hossein Antikchi, Hossein Neamatzadeh

**Affiliations:** 1 Department of Colorectal Surgery, Imam Reza Hospital, AJA University of Medical Sciences, Tehran, Iran; 2 Department of Internal Medicine, Yazd Branch, Islamic Azad University, Yazd, Iran; 3 Hematology and Oncology Research Center, Non-Communicable Diseases Research Institute, Shahid Sadoughi University of Medical Science, Yazd, Iran

**Keywords:** machine learning, gastric cancer, SEER database, prognostic models, data quality, precision oncology

## Abstract

The Surveillance, Epidemiology, and End Results (SEER) database is widely used to develop machine learning prognostic models for gastric cancer, yet data limitations restrict their clinical utility. We critically appraise the recent multicenter machine learning study by Guan et al., which reported superior prognostication compared with TNM staging, and identify eight methodological concerns: chemotherapy sensitivity of only 68% with systematic under capture of one-third of treated patients; complete loss of regimen-specific information that prevents distinction between curative perioperative therapy and palliative regimens; absence of performance status data despite it being the strongest predictor of outcome; missing molecular biomarkers (HER2, MSI-H, TMB, TCGA subtype) increasingly essential for precision oncology; unknown surgical technique quality metrics; and substantial cohort heterogeneity (surgery rates 26.6% vs 59.8%). These limitations collectively prevent individual treatment selection at the bedside. We conclude that SEER-based machine learning models should remain complementary to—rather than substitutes for—guideline-based decision-making, and future progress requires integration of regimen-specific treatment data, documented performance status, comorbidity indices, and molecular biomarkers.

## Main Text

**Fig. 1:**
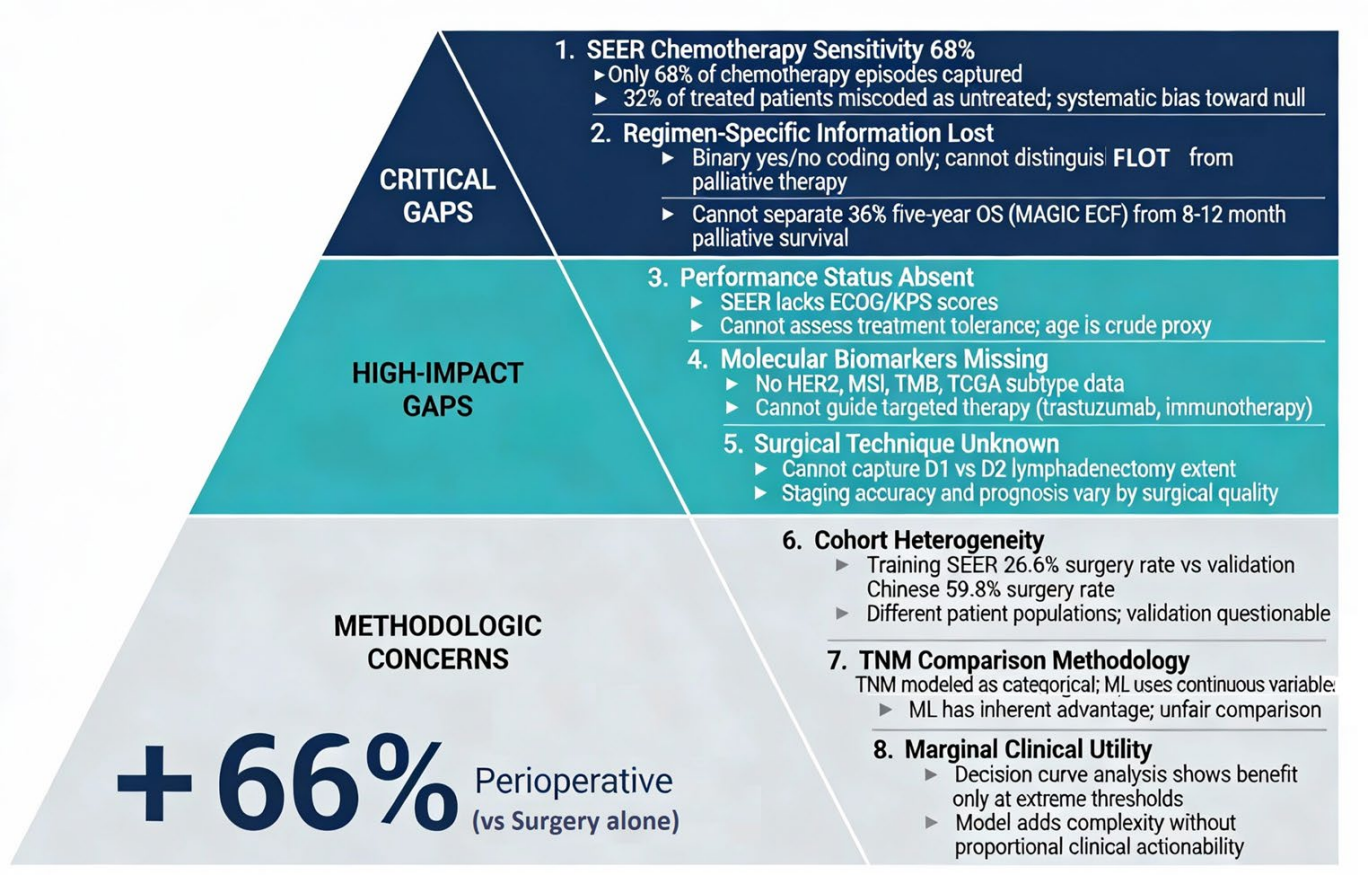
Eight data limitations of SEER-based machine learning models in gastric cancer organized by clinical impact severity. Tier 1 (Critical Gaps): chemotherapy capture sensitivity 68% [2]; loss of regimen-specific information conflating perioperative ECF (36% 5-year OS) [3] with palliative regimens (8–12 months) [6]. Tier 2 (High-Impact Gaps): absent performance status [7,8]; missing molecular biomarkers (HER2 [10], MSI-H [11], TMB, TCGA subtype [9]); unknown surgical technique [12]. Tier 3 (Methodologic Concerns): cohort heterogeneity [1]; TNM comparison methodology; marginal clinical utility

**Fig. 2:**
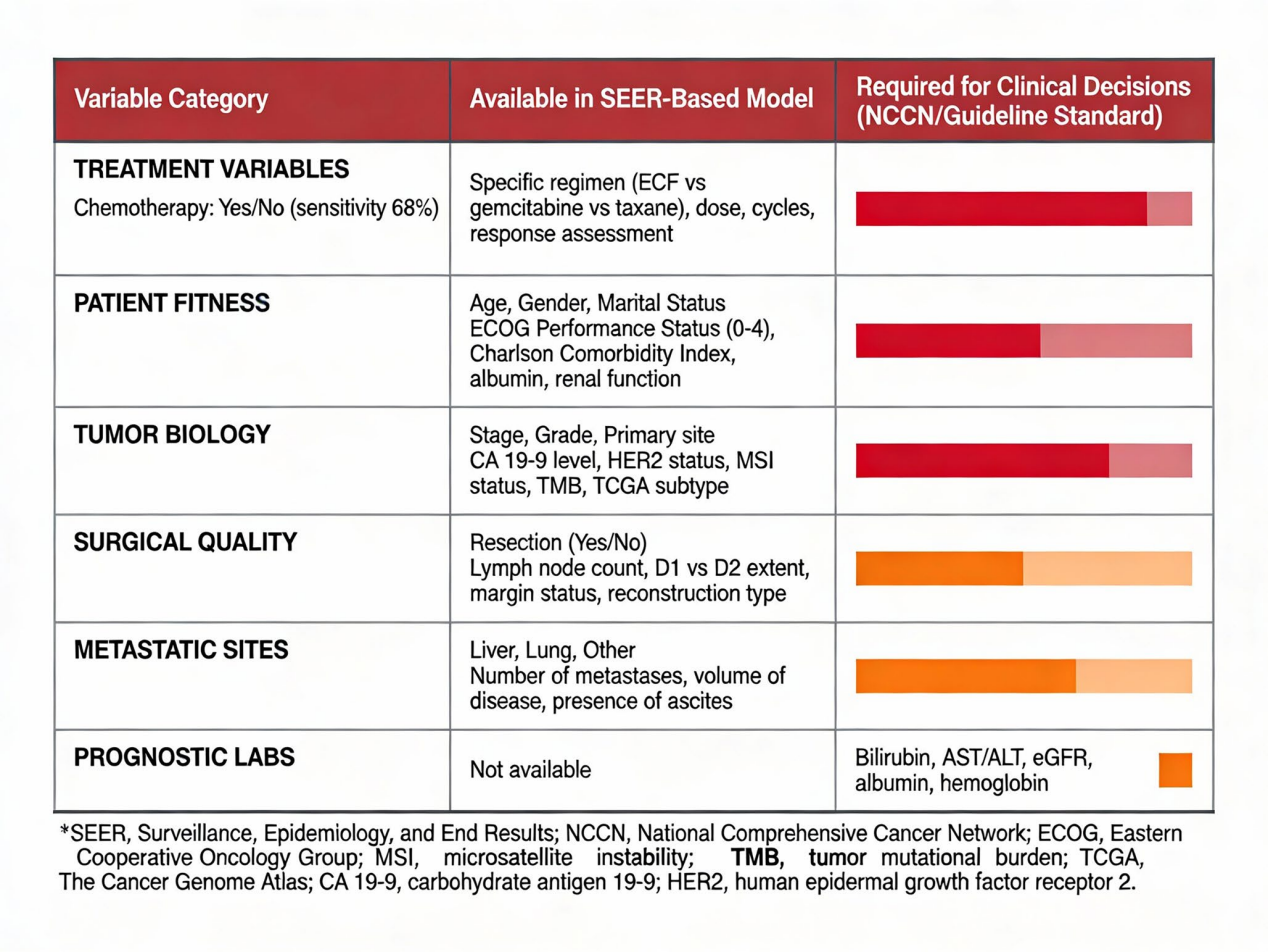
Comparison of SEER-available variables (left column) versus NCCN guideline-required information (right column) for gastric cancer treatment decisions [8,12]. Treatment: binary chemotherapy (68% sensitivity) [2] versus regimen specifics [3,4]. Patient fitness: demographics versus ECOG status, comorbidity indices, labs [7,8]. Tumor biology: stage/grade versus HER2 [10], MSI [11], TMB, TCGA [9], CA 19 − 9 [12]. Surgical quality: resection status versus D1/D2 extent [12], margins, reconstruction. Metastatic sites: organ only versus burden metrics. Prognostic labs: not available versus bilirubin, AST/ALT, eGFR, albumin, hemoglobin. Color intensity: red (critical), orange (high-impact)

We read with considerable interest the recent multicenter study by Guan et al. [1] published in Langenbecks Archiv für Chirurgie, which developed and validated machine learning–based survival prediction models for patients with gastric adenocarcinoma. The authors reported their integrated ensemble model achieved concordance indices (C-index) of 0.693 for overall survival (OS) and 0.719 for cancer-specific survival (CSS) using the Surveillance, Epidemiology, and End Results (SEER) database combined with data from two Chinese medical centers. While this represents a valuable effort to leverage population-level data for prognostic insight, limitations in SEER treatment variables—especially chemotherapy recording—restrict the model’s clinical applicability for guiding individual treatment decisions. Figure 1 illustrates these limitations organized by clinical impact severity, and Fig. 2 compares SEER-available variables with clinically required information.

## Chemotherapy data incompleteness in SEER

The most significant limitation is the well-documented low sensitivity of SEER chemotherapy data. The National Cancer Institute reports that, when SEER records are compared with Medicare claims, chemotherapy sensitivity is only 68%, with positive predictive value (PPV) exceeding 85% [2]. This asymmetry means SEER reliably records chemotherapy when given but systematically misses approximately one-third of patients who actually received treatment.

Consequently, the binary “chemotherapy: Yes/No” variable in Guan et al.‘s model conflates two fundamentally different groups: untreated patients and treated patients with missing data. This measurement error biases the estimated treatment coefficient toward the null. More importantly, clinicians rely on chemotherapy sensitivity data when interpreting predictive models, yet SEER-based models cannot quantify how this 68% capture rate affects predicted treatment benefit—a gap in model transparency that limits clinical interpretation.

## Loss of regimen-specific prognostic granularity

Even when captured, SEER codes chemotherapy as a binary variable. Modern gastric cancer management, however, depends entirely on specific regimen selection. The landmark Medical Research Council Adjuvant Gastric Infusional Chemotherapy (MAGIC) trial demonstrated that perioperative epirubicin, cisplatin, and fluorouracil (ECF) achieves 5-year overall survival (OS) of 36% versus 23% with surgery alone (hazard ratio [HR] 0.75, 95% confidence interval [CI] 0.60–0.93; *p* = 0.009) [3]. The Southwest Oncology Group (SWOG) 9008/Intergroup trial 0116 (INT-0116) showed that adjuvant chemoradiotherapy improves median overall survival to 36 months compared to 27 months with surgery alone (*p* = 0.005) [4, 5]. By contrast, palliative chemotherapy for metastatic disease yields median survivals of 8–12 months [6].

In the Guan model, these vastly different clinical scenarios are indistinguishable—all coded as “chemotherapy: Yes.” A 50-year-old fit patient receiving perioperative ECF with potential for 36% 5-year overall survival and a 70-year-old with metastatic disease receiving single-agent palliative therapy (median 10 months) would be treated identically by the model’s chemotherapy variable. This incompatibility with modern regimen-specific practice represents a fundamental data granularity gap. The model cannot answer the clinical question that matters: Which regimen benefits which patient?

## Absence of performance status and comorbidity assessment

Performance status (Eastern Cooperative Oncology Group [ECOG] or Karnofsky) is the single strongest predictor of overall survival (OS) and treatment tolerance in gastric cancer, with magnitude of effect often exceeding Tumor, Node, Metastasis (TNM) stage [7, 8]. The SEER database does not capture performance status. Consequently, Guan et al.‘s model relies on weaker proxy variables—age, gender, socioeconomic factors—to infer physiologic reserve, introducing substantial confounding.

Consider two patients: a fit 75-year-old (ECOG 0) versus a medically frail 50-year-old (ECOG 3). The fit older patient tolerates intensive perioperative chemotherapy; the frail younger patient cannot. Yet the model would likely assign superior survival predictions to the younger patient based on age alone. This reversal of clinical reality demonstrates how absence of performance status data undermines the model’s clinical validity. SEER also lacks formal comorbidity indices, nutritional markers (albumin), and organ function data—all essential determinants of treatment tolerance and outcome.

## Missing molecular and biomarker information

Precision oncology in gastric cancer increasingly relies on molecular stratification. The Cancer Genome Atlas (TCGA) identifies four distinct subtypes—Epstein–Barr virus-associated, microsatellite instability-high (MSI-H), genomically stable, and chromosomal instability—each with different prognoses and treatment implications [9]. Human epidermal growth factor receptor 2 (HER2) status is an essential predictive biomarker; the Trastuzumab for Gastric Cancer (ToGA) trial demonstrated that HER2-positive patients benefit from trastuzumab combined with chemotherapy (median overall survival [OS] 13.8 months vs. 11.1 months; HR 0.74, 95% CI 0.60–0.91; *p* = 0.0046) [10]. Microsatellite instability-high (MSI-H) status predicts response to immune checkpoint inhibitors, now increasingly deployed in gastric cancer [11]. Preoperative carbohydrate antigen 19 − 9 (CA 19 − 9) level provides independent prognostic value [12].

None of these biomarkers are available in SEER, and therefore absent from Guan et al.‘s model. As precision medicine advances and targeted and immunotherapies become standard, prognostic models lacking molecular granularity risk misalignment with real-world treatment pathways. The model cannot incorporate HER2 status to guide trastuzumab decisions, microsatellite instability or tumor mutational burden (TMB) status to guide immunotherapy, or other increasingly important biomarkers.

## Surgical technique and cohort heterogeneity

Lymphadenectomy extent (D1 vs. D2) materially affects Tumor, Node, Metastasis (TNM) staging accuracy and survival outcomes [12]. Current National Comprehensive Cancer Network (NCCN) guidelines recommend D2 lymphadenectomy in experienced centers [12]. SEER does not reliably capture lymphadenectomy extent or surgical quality metrics, limiting international validation where surgical practices differ substantially.

Furthermore, training and validation cohorts reveal notable differences. The SEER cohort (*n* = 21,559, surgery rate 26.6%) and Chinese cohort (*n* = 3,805, surgery rate 59.8%) differ substantially—a 33%-point gap [1]. These differences likely reflect variations in stage distribution and patient selection between population-based registry data and single-center Chinese cohorts [1], raising questions about model generalizability. Without detailed stratification, implications for external validity remain unclear.

## Implications for clinical use

Taken together, these limitations indicate that the Guan model is best understood as a tool for population-level risk stratification rather than for individualizing treatment decisions. It may identify very-high-risk or very-low-risk cohorts for surveillance discussions, but cannot answer: Should this patient receive perioperative chemotherapy or adjuvant chemoradiotherapy? Is this patient fit for intensive regimens? Which biomarker-directed therapies apply?

The model’s reported superiority over TNM staging warrants careful interpretation. Tumor, Node, Metastasis (TNM) was modeled as categorical, while the machine learning model leveraged continuous covariates—conferring an inherent statistical advantage. Decision-curve analyses typically show only marginal net benefit over TNM within clinically relevant probability ranges. Until SEER-based models incorporate regimen-specific treatment data (ideally via electronic health record [EHR] or Medicare claims linkage), documented performance status, formal comorbidity indices, and key molecular biomarkers, their role should remain complementary to guideline-based clinical decision-making.

## Questions for the authors

1. Can you provide sensitivity analyses demonstrating how variations in chemotherapy capture sensitivity (50–75%) affect the chemotherapy coefficient magnitude and statistical significance?
2. Would external validation in a prospectively collected cohort with complete performance status, chemotherapy regimen details, and molecular biomarker data be feasible?
3. How do you envision clinicians would apply this model to select specific chemotherapy regimens given the binary chemotherapy coding in SEER?
4. Do you view this model as appropriate for individualized treatment selection, or primarily as a population-level prognostication tool?

## Conclusion

Machine learning offers promise for leveraging large-scale registries to advance gastric cancer prognostication. The study by Guan et al. makes a valuable contribution. However, SEER’s well-established limitations in chemotherapy sensitivity (~ 68%), complete absence of regimen specificity, lack of performance status and comorbidity data, and missing molecular biomarker information substantially constrain the model’s utility for treatment decision-making. Future progress requires integration of regimen-specific data, performance status assessment, and molecular biomarkers. Until these comprehensive data are available, clinicians should interpret SEER-based machine learning models as complementary to—rather than substitutes for—guideline-based decision-making informed by landmark trials (MAGIC, SWOG 9008, TOPGEAR, CRITICS).

## Data Availability

Not applicable. This analysis uses published literature from PubMed, PubMed Central, and the National Cancer Institute SEER database.
